# Mr. Scott on Tic Douloureux and Neuralgia

**Published:** 1835-01-01

**Authors:** 


### Cases of Tic Douloureux, and other Forms of Neuralgia. By *John Scott*, Surgeon to the London Hospital, &c. Octavo, pp. 52. Longman and Co. Oct. 1834.

The whole class of neuralgiæ is a most deceitful one—and tic douloureux is a veritable ignis fatuus. It may not, indeed, as in the days of the merry monarch—

"Lead men into pools and ditches,"

but it certainly leads medical practitioners to draw erroneous conclusions—especially when the inferences are flattering to themselves. We are always ready and willing to give new remedies a fair trial, and new doctors a patient hearing; but sad experience has repeatedly convinced us, that we are often too credulous in the one case and too indulgent in the other. Nevertheless, we are still inclined to believe that we err on the right side—and that it is better to let half a dozen suspicious characters pass into the first ordeal of probation, under a lenient scrutiny, than to repulse one honest candidate, whose claims are of a doubtful character. In respect to the disease under consideration, we are willing to believe that many candid practitioners have been deceived themselves, rather than inclined to deceive their brethren. A few, or even a series of cases of neuralgia do well under a peculiar treatment, and then they fondly and naturally conclude that they have hit the mark, and made a discovery. This is the case in all obstinate and nearly incurable diseases. No one can suppose that Dr. Hutchinson was not sincere, when he set forth carbonate of iron as nearly infallible in tic douloureux. The carbonate proved useful in a very small proportion of cases. Dr. Turnbull was still more confident with his veratria, and, if we may judge by our own experience, this remedy will not prove nearly so beneficial as Dr. Hutchinson's specific. Mr. Scott, the author of the work before us, has come forward with respectable claims to attention. He is surgeon to a large metropolitan hospital—he is the son of a medical practitioner, whose fame has resounded from pole to pole—and we have heard (for he is personally unknown to us) that he is a gentleman of probity and veracity. We are, therefore, bound to listen to his reasonings and to credit his statements, provided the former are just and the latter uncontroverted. The degree of faith which we may place in the proposed remedies, and the nature of the anticipations which we may have formed respecting the result, are matters of private consideration, and ought not to appear on the analytical record of unbiassed journalists. The preface is so unusually short, that we can give it insertion.

"The local treatment described in the following pages has afforded such decided relief in a considerable number of cases which had resisted all other remedies, that I feel it incumbent on me to communicate a few of them to the profession, that its efficacy may be tried on a more extended scale.

In doing this, I have felt anxious to contrast the description of cases which may be thus relieved with others that require very different treatment, that I may not appear to fall into the too common error of adopting the same mode of practice under dissimilar circumstances, or be understood to recommend the indiscriminate employment of any remedy in a disease so various in its causes as Tic Douloureux."—*Advertisement.*

We shall not dwell on Mr. Scott's definition and description of tic douloureux—the former being seldom possible in any disease, and the latter, in the present case, being but too well known. Mr. Scott makes a part of his definition—*the absence of any disease in the surrounding structures.* This is somewhat bold. He limits the term "tic douloureux" to an affection of one or more nerves of the face. It is not always unattended by disease of neighbouring structures—nor is it peculiar to the nerves of the face. We have seen it as exquisitely marked in various parts of the body—even in the foot, as in the face.* Mr. S. is much too positive. He says the paroxysm of pain "is as severe at its commencement as at any period of its duration." Every practitioner must have seen instances, where the acmé of pain was some time after the beginning of the attack. Mr. S. might have avoided criticism, in these instances, by using the word *generally* instead of always. The wider our range of experience is, the more cautious we become of the term "*always*," as applied to the definition or description of human infirmities.

"Notwithstanding the intensity of the pain, there is neither increased heat nor redness of the soft parts; and in the early stages of the disease, the nerve is not tender to the touch in the interval between the paroxysms, although it is exquisitely so during their continuance: when, however, the disease has been of long standing, *tenderness of the nerve is a marked and constant symptom;* and then, usually, the slightest touch or movement of the part will induce a paroxysm." 3.

Can such marked tenderness in the nerve, on the slightest movement or touch of the part, indicate less than some change in the structure? We apprehend not. Why should this tenderness be absent in the beginning, and present in the advancement of the disease? It requires no great pathological knowledge to solve this question. There is much truth, and, we believe, some error, in the following passage.

"The progress of the disease is generally marked by an increase in the severity, duration, and frequency of the attacks; so much so, as at length scarcely to allow any interval of rest to the afflicted sufferer between the periods of their recurrence. Sometimes the paroxysms will return with such certainty and severity, when the patient is warm in bed, that he anticipates with terror the hour of their accustomed access, and passes whole nights in an easy chair: in other cases, the pain is relieved when he is warm in bed, but it is intense during the day, induced by the slightest muscular action, or even by a breath of air, or the slightest touch. Thus he dreads the hour of meals, knowing that mastication will surely renew the paroxysm with great severity. Sometimes, even mental excitement will induce an attack, and often it will occur spontaneously, without any assignable cause whatever, and, generally speaking, these attacks are more severe and of longer continuance than those which result from local exciting causes. Sometimes the pain is regularly intermittent, recurring daily at the same hour, and this more frequently in the evening than in the morning; and after remaining a longer or a shorter time, it disappears entirely until the usual hour of its recurrence on the following day. In other cases, you have two or even three attacks in the twenty-four hours, coming on regularly at stated pe-

---

* Mr. Scott himself quotes a case from Chaussier, where tic douloureux of the plantar nerve alternated with the same affection of the infra-orbitar branch of the fifth pair. *See page* 8.

riods. *Whether the disease be thus periodical in its access or not, when it has been of some time standing, the pain does not altogether disappear between the paroxysms.* The intervals are marked only by a remission of the symptoms, but attended with constant tenderness and distressing uneasiness in the part, which is, perhaps, confined to a spot of the size of a silver three-pence, and renders it liable to the production of a spasm on the application of the most trifling exciting cause." 5.

Now we know several cases, and, if we are not much deceived, Mr. Scott knows of one or two cases, where the Tic, even of the face, has been of long standing (if ten or a dozen of years may be considered as such), and where there are intervals of complete immunity from the pain. Here is another instance where the author is incautious, and where "*generally*" would have been a useful word in descriptions.

Mr. Scott justly remarks, that such intense and protracted sufferings usually induce secondary or constitutional symptoms after a certain period; such as impaired strength—disordered digestion—and irritability of temper.

"Tic Douloureux has been divided into four species, designated according to the situation of the nerve that is the seat of this affection.

The first species, or that affecting the supra-orbitar branch of the fifth pair of nerves, is characterised by pain, which usually commences at the exit of the nerve from the supra-orbitar foramen, and extends chiefly over the forehead, eyebrow, and upper eyelid on the affected side, which is generally closed during the paroxysm : the pain involves the globe of the eye itself, it is attended with throbbing of the neighbouring arteries, and tension of the corresponding veins, together with a copious flow of tears.

Sometimes the pain proceeds in the opposite direction deeply into the orbit, along the trunk of the affected nerve, involving also the surface of the eye, which itself becomes somewhat bloodshot during the paroxysm, and this injection of the conjunctiva is often rendered permanent after repeated attacks ; sometimes it is attended with a dull obtuse pain in the frontal sinuses, with dryness and irritation of the mucous membrane lining the nares.

The second species, or that affecting the infra-orbitar branch of the fifth pair of nerves, though often sudden in its access, sometimes comes on more gradually, being preceded by a pricking, tickling sensation at the side of the cheek, and spasmodic action of the lower eyelid ; these symptoms are shortly succeeded by pain at the infra-orbitar foramen, extending over the cheek as far as the zygoma, affecting the lower eyelid, ala nasi, and upper lip, and often terminating abruptly at the mesial line of the face; sometimes it extends to the teeth, the antrum, the hard and soft palate, even to the base of the tongue, and induces violent spasmodic contractions in the muscles.

In the third species, or that affecting the inferior maxillary branch of the fifth pair of nerves, the pain originates at the orifice of the mental foramen ; extends to the lips, the alveolary processes, the teeth, the soft parts beneath the chin, and to the side of the tongue, and very rarely it extends along the dental canal towards the origin of the nerve. The pain often ceases abruptly, precisely at the symphisis of the chin ; but it frequently extends to the whole cheek as high as the malar bone, and even the ear. The general expression of the features, too, is much distorted during the paroxysm by spasmodic action of the muscles of the face, and this to such an extent as to throw them into a violent tetanic spasm, holding the jaw fixed and motionless during its continuance.

The fourth species has been usually described as affecting the facial nerve ; *but since this has been demonstrated, by recent experiments, to be exclusively a nerve of motion, it seems scarcely possible that it can be the seat of the disease in question;* and I have generally considered that those cases which have been classed under this head, on account of the situation of the pain, were, in fact, affections of some of the branches of the fifth pair of nerves." 8.

If Mr. Scott has ascertained that tic douloureux is confined to nerves of sense, and incapable of affecting nerves of motion, he has arrived at a pitch of knowledge which we are unable to contemplate. Experiments may seem to prove that the facial nerve is *exclusively* a motor nerve, and that the plantar nerve may have two roots, one for sensation, and the other for motion; but we apprehend that *experience* will often modify, if not nullify, *experiment*. How often do we find parts supplied by the ganglionic nerves, and which are usually devoid of common sensation, become acutely sensible in a state of disease? Who, while, in health, feels any uneasiness from food in the stomach? Who, that has dyspepsia, is free from pain during the process of digestion?

The metastasis, however, does not always relieve the primary seat of the pain. It sometimes appears to send a portion of its power to a distant spot, keeping possession of its accustomed habitat at the same time. It is equally uncertain in its duration and period of recurrence, as in its seat.

"I have at present two gentlemen under my care, who are frequently seized with violent paroxysms of pain in various parts of the lower limbs, which manifests no constancy in its seat or duration, scarcely ever occurring twice successively in the same spot; sometimes it will remain fixed during the whole paroxysm in one situation, and at other times it will affect various parts of the limb rapidly in succession, or become instantly transferred to the opposite limb. In one of these cases the external use of veratria, in the manner recommended by Dr. Turnbull, gives relief to the spasms, but of course it does not prevent their recurrence; the other has yielded to constitutional treatment." 10.

The above passage proves the error of Mr. Scott's definition by his own words. Some forms of this complaint, Mr. S. observes, are frequently mistaken for toothache, and many teeth are thus uselessly sacrificed. Mr. S. adverts to the two principal opinions respecting the nature of TIC DOULOUREUX—one, that it is caused by preternatural vascularity of the neurilema—the other, that it depends on disturbed function of the nerve itself, without any physical change in the part. Parry, Cirillo, Chaussier, Delpech, Rousset, Swan, and many others, incline to the first theory—and Mr. Scott himself is of this party, and argues for the inflammatory nature of the disease.

"Tic douloureux, as well as the other forms of neuralgia, appear almost invariably to originate in some constitutional source of irritation to the nervous system; those cases excepted, in which it arises from a nerve being irritated by diseased or decayed bone in its vicinity, or those in which it is caused by a wound of a nerve, or depends on the presence of a foreign body, as a bullet lodged in the part.

It may, however, be a question, whether these local causes are capable of producing this affection in a perfectly healthy person free from any morbid condition of the nervous system. We constantly meet with cases in which the bones are the seat of simple or specific disease—the superior and inferior maxillary bones, for example, through which the nerves so liable to this affection have their course—without the disease in question being produced. Wounds and laceration of nerves, and the lodgement of foreign bodies in their vicinity, are so rarely followed by this complaint, that its occurrence must be regarded as an exception to the general rule." 15.

Mr. S. relates a case of a lady who had a constant and distressing pain in the right arm, extending from the bend of the elbow down to the fingers. It arose from the operation of bleeding, the cicatrix of which was evident; and the slightest pressure on this point gave intense pain that thrilled down to the thumb and some of the fingers, "leaving no doubt that the median nerve had been wounded." The patient was of a highly nervous temperament, bordering on mental derangement. In addition to constitutional remedies, he employed the local treatment hereafter described, and a cure was effected in the course of a month. Mr. S. arranges the consitutional causes of tic doloureux under the five following heads:—

1. "A plethoric state of system, which, however, it must be observed, very rarely gives rise to the affection.

2. An asthenic state of system, which certainly produces the disease much more frequently than the opposite condition; hence its frequent occurrence in the decline of life, when the power of the circulation begins to fail, and during periods of mental suffering and anxiety.

3. A gouty or rheumatic diathesis seems particularly liable to the disease, which often occurs in conjunction with affections of this character in other textures, or immediately on their subsidence; and it is observed that persons who are habitually exposed to cold and moisture, as fishermen, and the inhabitants of marshy districts, are particularly liable to the disease in question.

4. A disordered condition of the digestive organs, attended by the usual symptoms which characterise such derangement.

5. The impression of malaria on the system; in which case the disease assumes the intermittent character, the paroxysms recurring regularly at a given hour, and increasing or lessening in duration as the disease becomes more or less severe." 17.

In the plethoric cases, the treatment must, of course, be directly the reverse of that which is necessary in the asthenic constitution. It is in this last form that the carbonate of iron, Mr. S. thinks has been often attended with the most favourable results. The same remark is applicable to bark, arsenic, &c. Two cases are related where the pain was periodical, and the patients weak. The carbonate of iron was successful in one case, but it required quinine and other means in the second. The veratria in this instance gave little or no relief. We have, indeed, been greatly disappointed during the last few months, in this medicine, and suspect that it will go to the "Tomb of the Capulets," ere many years have rolled away.

"Under this, the second head (asthenic) of the disease, may be classed that irritable condition of the brain and nervous system to which some persons, especially delicate females, seem to be extremely prone when their constitutional power is reduced below the natural standard,—a state extremely liable to the disease in question, and one in which hemlock seems to be productive of very considerable benefit, when exhibited in the manner long ago recommended by Dr. Fothergill." 22.

According to our author's experience, a gouty or rheumatic diathesis is far more frequently productive of TIC than any other constitutional cause—and when this is the case, "it will often yield, with surprising rapidity, to those remedies which correct this morbid condition of the system." The following case is given as a striking example.

*Case.* "In Nov. 1828, I visited a gentleman at Hampstead, labouring under one of the most severe forms of the disease I ever witnessed, affecting the first branch of the fifth pair of nerves on the right side. The pain commenced with a falling of the eyelid, and corrugation of the brow, attended with a throbbing sensation in the part; which symptoms indicated the approach of the attack. It came on gradually, commencing at sunrise; became very severe about ten o'clock in the morning; and at noon the pain would be often so excruciating, that he could neither sit, stand, nor walk, but actually writhed in agonies on the carpet, and was obliged to go to bed to hide his sufferings; from noon, the pain gradually decreased in severity until the evening, when it entirely subsided; so that he slept perfectly well during the early part of the night, and was awoke in the morning by the renewal of his sufferings. The attacks were more violent in cold, particularly during an easterly wind, than in warm weather; but they always affected the same spot, occurred at the time and in the manner above described, and pursued their own course, without being relieved by any remedies, or aggravated by any exciting causes: neither mastication nor talking would produce them, nor increase their severity; but at one time they certainly appeared to be much more violent during a period of great mental anxiety. These attacks would recur daily for two or three weeks, and then spontaneously subside for two or three months, and again recur without any assignable cause; at other times they would occur regularly for a day or two, and then there would be an interval of a week or more before any fresh attack: in short, there was no regularity in the frequency of their recurrence, nor in the length of time they would continue; but he never passed a year without many attacks, for a period of twenty-eight years, with the exception of three successive years; during the whole of which he was subject to constant attacks of acute rheumatism, in almost every joint in the body in succession, and they were so severe, of such continuance, and so frequently renewed, that he scarcely left the house during that period; but he was perfectly free from Tic Douloureux the whole of this time. As soon, however, as the rheumatism disappeared, the original disease was reproduced with its usual frequency and severity. Of course, a vast variety of treatment was adopted at various times: among other remedies, he took large quantities of quinine of iron and of colchicum, but nothing appeared to have the slightest influence on the disease; blisters, leeches, opiates, &c. &c. were employed, and he was advised to have the nerve divided, but would not consent to the operation.

Finding, from the history of the case, that the disease evidently depended on a rheumatic diathesis, which I observed was kept up by an unhealthy condition of the mucous membrane of the alimentary canal, and of the hepatic secretions, evidenced by a peculiarly white tongue, and by the nature of the evacuations: instead of attempting to act specifically on the affection, I at once proceeded to correct this faulty disposition of the system, and with this view prescribed the following medicine:—

℞. Hydr. Submur. gr. ij.
Extract. Coloc. comp., Pulv. Scammon., āā. gr. v. M. et divide in pilulas ij. alternis noctibus sumendas.

℞. Extracti Sarsaparillæ, ℥ss. Aquæ distillatæ, ℥vj. M. ft. mistura, cujus capiat quartam partem sextâ quâque horâ.

Under this mode of treatment the secretions rapidly improved; the tongue became gradually clean; the flatulence, which before was very distressing, disappeared; the appetite, which had been much impaired, returned; and his health and strength became manifestly recruited; and in proportion to the improvement which thus occurred in the state of his constitution, did the virulence of the disease gradually abate, and in three weeks it wholly disappeared. He was of course directed to pay particular attention to the state of his general health, and above all things to preserve the utmost regularity in the action of the bowels; and by so doing he has remained free from the slightest return of the complaint up to the present time." 25.

When the disease arises from irritation of the nerves, dependent on disordered digestion, it will repeatedly disappear on restoration of healthy action in the chylopoietic organs, provided that this be done before the disease has become established.

When the disease assumes the intermittent form, and arises from malaria, the remedies of ague are generally efficient. The following passage is highly commendable.

"From the foregoing concise review of the nature of Tic Douloureux, and the constitutional causes in which it originates, it must be obvious that, if any one expects to remove a disease depending on such various circumstances and such opposite conditions of the system by any specific remedy, however powerful, his expectations must issue in disappointment in a vast majority of cases. It is only by a patient investigation of the peculiar circumstances attending each particular case, that we can ascertain the causes on which this or any other disease may depend; and until they are removed, all attempts at its alleviation must be utterly futile.

It seems, however, that most of the remedies that have been so confidently announced as having the power of curing the disease in question, have been recommended and employed without that discrimination which is quite as essential to the successful treatment of the affection as the power of the remedies themselves." 29.

This brings us nearer the immediate object of the work—the local treatment. Our author observes that, although the disease, at its commencement, depends on constitutional causes, the removal of which will generally remove the complaint, yet, if not arrested in the early stages, the local manifestation will continue unchecked by constitutional remedies, and consequently requires particular local management. He properly guards against the inference that this local treatment should be indiscriminately employed in every case.

"Some years ago, when I was trying the efficacy of the local influence of mercury in arresting chronic inflammation of the various structures in the body, with a view to illustrate the treatment of diseased joints on this principle, I met with several very obstinate and protracted cases of Tic Douloureux, which had resisted every remedy that could be devised, and I determined to subject them to that mode of treatment which I had found so successful in what I believed to be a similar condition of other textures.

The plan I adopted was to keep constantly applied to the part, on a piece of flannel, an ointment composed of one drachm of tartarised antimony and an ounce of mercurial ointment, renewing it as frequently as it could be borne, the object being to produce such a degree of irritation on the skin as would insure the mercurial influence on the part.

This method was attended with very considerable success, but I have not preserved any record of the cases in which it was employed at this time." 31.

An interesting case is here recorded, of an Essex man, 62 years of age, who consulted our author in February, 1829, for an agonizing form of tic in the face. The paroxysms were momentary, recurring suddenly every few minutes, by day and by night, the intervals being free from pain. The paroxysms were, at first, brought on by the act of deglutition—afterwards, by mastication or articulation, and this with great severity, so that he was obliged to give up speaking altogether. He had been afflicted, more or less, for 13 years. He was subject to rheumatism, and of rather a full habit. Almost every remedy had been tried and failed. After giving some aperient pills, the hydrargyro-antimonial ointment was applied to the face on a piece of flannel. It produced great irritation, but in a fortnight the paroxysms were decidedly relieved. In a month he was well. He lived till 1833, without return of the complaint.

"It subsequently occurred to me, that the iodurets of mercury, particularly the deuto-ioduret, would be a much more effectual remedy than the preceding application; producing more speedily such an abrasion of the skin, as would subject the part to the mercurial influence; and the following are cases in which this mode of treatment has been adopted,—using the deuto-ioduret, the proto-ioduret of mercury, or the common mercurial ointment, according to the state of the skin; the object being to produce such an effect upon the part as would control the disease, and to keep up the impression of the remedy to such an extent, and for such a length of time, as would remove the morbid condition of the nerve." 34.

Several cases in illustration are detailed. In the first case, a very desperate one, an ointment composed of two scruples of the deuto-ioduret of mercury, well mixed with an ounce of lard, were rubbed on the affected side of the face night and morning, until the irritation could be no longer borne. The pain was soon relieved; but afterwards returned, because the counter-irritation was too soon left off. She therefore recommenced the use of the ointment; and in a month she was quite well. "She had now lost the aged look and expression of anguish in her countenance, which had been induced by such severe and protracted sufferings, and her general health was greatly recruited."

We shall quote the following case entire, because we know the patient well, as we have attended the family for eight or ten years past. The lady herself was generally attended in her long and reiterated sufferings, by Dr. Kerrison.

"On the 23d of September, 1833, I visited a lady in Albemarle Street, who was labouring under a very severe attack of Tic Douloureux in the supra-orbitar branch of the fifth pair of nerves on the left side. I was informed that she had been subject to repeated attacks of the disease for the last eight years, after intervals of six months, a year, and eighteen months, sometimes in its present situation, sometimes in the lower jaw; that she was of a gouty family, and had been herself repeatedly subject to hepatic derangement.

The attack on account of which I was consulted had been of a month's continuance: the paroxysms were brought on by eating, by talking, by any motion of the face, and even by any mental excitement; they occurred also spontaneously, very often during the day, and invariably as soon as she lay down in bed at night.

The paroxysms came on instantaneosly, without any previous warning; they lasted with the utmost intensity for ten or fifteen minutes, and then completely subsided as suddenly as they commenced, leaving neither pain nor tenderness in the part, although it was exquisitely sensitive during their continuance.

The attacks had been gradually becoming more frequent in their recurrence, as well as increasing in their severity, notwithstanding that the most judicious treatment had been adopted for their alleviation, and for the preceding twenty-four hours they had been almost incessant, day and night. Such severe and protracted agony had necessarily induced great prostration of strength and excessive nervous irritability, and this too was aggravated by the ineffectual efforts to relieve her sufferings by the use of opiates.

I directed the opium and all other remedies to be discontinued, and applied the deuto-ioduret of mercury spread on lint to the part affected. This very shortly produced vesication of the cuticle, and its effect on the disease was instantaneous; for in the first twenty-four hours after its application, she experienced only three paroxysms of pain, whereas they had been previously incessant; on the following day they were rather more frequent, and continued to recur occasionally in a very slight degree during the three succeeding days, after which they ceased altogether. The ointment was renewed as frequently as the skin would bear its application, and was varied according to the effect it produced; sometimes the proto-ioduret of mercury was employed, and sometimes the common mercurial ointment, so as to keep up the influence of the remedy on the part for a fortnight, when it was entirely discontinued.

This patient continued free from any recurrence of the disease until the 19th of January, 1834; when, after an attack of erysipelas, which was evidently induced by a very disordered state of the digestive organs, she was seized with very severe paroxysms of pain in the same situation as before. I immediately directed some medicine to be taken for the purpose of acting very freely on the bowels, which was to be repeated as frequently as it could be borne; the same ointment was also applied to the part, and it produced the usual vesication; but the paroxysms continued very severe during the following day and night, after which they entirely ceased. The medicine was not taken to the extent that had been desired; the effect of the ointment rapidly subsided, and the consequence was a recurrence of the paroxysms on the succeeding Sunday, the 26th.

On the following day the patient came to town; the ointment was again applied; and as soon as its usual effect was produced upon the skin, the paroxysms were decidedly influenced, and gradually disappeared; indeed, she felt very little of them until Friday, the 2d of February, when, in consequence of the effect of the application having been allowed altogether to subside, they returned with considerable severity.

The ointment was immediately re-applied, and again checked the disease, two or three paroxysms only being experienced during the three succeeding days, after which they entirely ceased. Although the disease was thus decidedly influenced by the remedy on each time of its application, as soon as the effect was allowed to subside, it immediately recurred, and this I attribute to the irritation the nervous system was labouring under at the time, from a very disordered condition of the digestive organs, which was evidently the exciting cause of the attack in question, as well as of the erysipelas which preceded it. Indeed, there was very considerable difficulty in obviating this disordered condition; the most active medicines being necessary, throughout the progress of the case, to promote a natural state of the secretions, which, for a very considerable time, resumed their former unhealthy appearance immediately on its being discontinued." 41.

We can state, on the authority of the lady herself, that her sufferings from the remedy so far exceeded any thing she had ever suffered from the TIC, that she would die rather than undergo the trial again. Mr. Scott has only to read this passage to Mrs. W——s, and convince himself of the truth of our statement.

The following passage concludes the work.

"From the view I take of the nature of the disease, it would appear that opiates should not be had recourse to, since they cannot relieve the morbid condition of the nerve in which the disease consists, and, therefore, are injurious, by increasing the irritability of the patient. To this rule, however, an exception presents itself in those cases, in which the disease is kept up by a morbid irritability of the brain and nervous system, over which hemlock seems to exert a beneficial influence. These instances, I am satisfied, would be rendered comparatively rare by pursuing the mode of treatment I have detailed; for although the state of the nervous system is such as to re-act upon and aggravate the local disease, this state is invariably kept up by the protracted sufferings of the patient, and will be more speedily and effectually relieved, and the patient restored to health and tranquillity, by removing the local source of irritation, than by the use of any narcotics, however powerful.

In those cases, then, in which the disease depends on local causes, it will yield to local treatment, and the subsidence of such constant pain will be speedily followed by a surprising renovation of the health; but where it is kept up by constitutional influence, instead of endeavouring to give relief by opiates, it is much more advantageous to alter the condition of the nerve in the manner I have mentioned, which will speedily alleviate the pain, at the same time that your attention is directed to the removal of the constitutional derangement, which otherwise would certainly induce a recurrence of the affection as soon as the local treatment ceased to be employed.

Of the efficacy of mercury in the local treatment of disease, I have elsewhere spoken at considerable length; therefore it is unnecessary again to enlarge upon the subject; and my experience enables me to speak with confidence of the combined effects of local and constitutional remedies in the treatment of neuralgic affections, which will often yield with great rapidity to their judicious employment. While, however, I am satisfied that the disease itself, as well as the acquired predisposition to its recurrence, may be thus so completely removed as to leave no danger of its spontaneous return, I would by no means be understood to assert, that any mode of treatment can alter the natural predisposition of the nervous system, and insure the patient from a reproduction of the complaint when exposed to the influence of exciting causes. If, therefore, a particular attention to the causes of the malady, in each individual case, is essential to the selecting of the most judicious mode of treatment for its relief, it is equally so in a prophylactic point of view; since it is only by a constant attention to this part of the subject that the recurrence of the disease is to be prevented." 52.

We have but few remarks to make. The work is written in a candid manner, and is perfectly free from mystery or exaggeration. Our experience of the disease, however, and our knowledge of one of the most remarkable cases introduced in the work, diminish very stronly any sanguine hopes from the *local* treatment forming the principal feature of the Essay. That such powerful and painful counter-irritation, as that employed by Mr. Scott, will occasionally supersede, for a time, the neuralgic suffering, we have no doubt; but beyond this, we have little faith in topical applications.

